# Supplementary material for: The causal relationship between the human gut microbiota and pyogenic arthritis: a Mendelian randomization study
Source: Front Cell Infect Microbiol. 2024 Nov 26;14:1452480. doi: 10.3389/fcimb.2024.1452480 (PMC11629706; doi:10.3389/fcimb.2024.1452480)
Supplement: Supplementary file 1 [file DataSheet1.zip › mendelian test/ebi-a-GCST90027615.csv_leave_one_out.pdf]

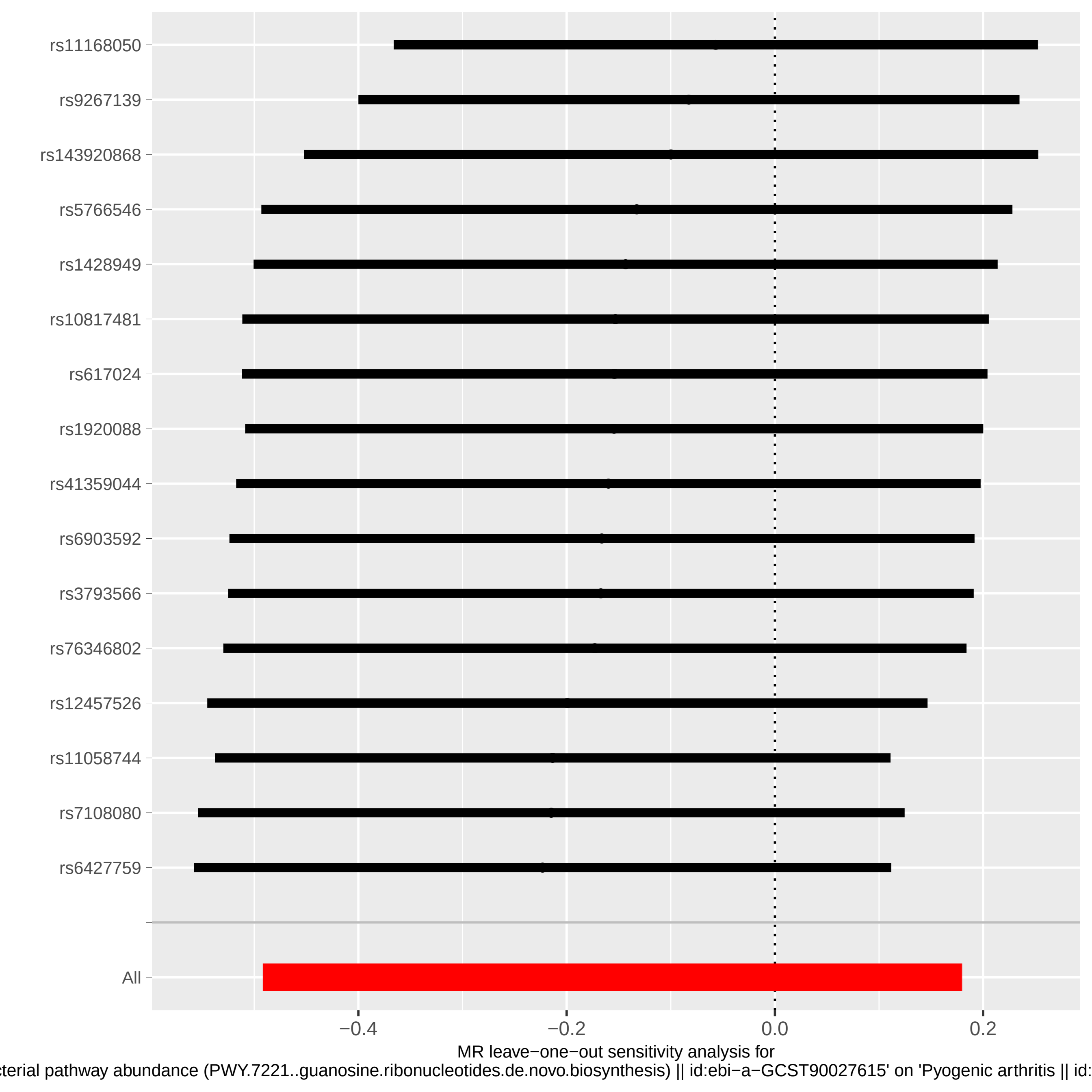

rs11168050
rs9267139
rs143920868
rs5766546
rs1428949
rs10817481
rs617024
rs1920088
rs41359044
rs6903592
rs3793566
rs76346802
rs12457526
rs11058744
rs7108080
rs6427759
All
−0.4
−0.2
0.0
0.2
MR leave−one−out sensitivity analysis for
cterial pathway abundance (PWY.7221..guanosine.ribonucleotides.de.novo.biosynthesis) || id:ebi−a−GCST90027615' on 'Pyogenic arthritis || id:
